# Clinical and Radiographic Evaluation of a Resin-Based Root Canal Sealer: 10-Year Recall Data

**DOI:** 10.1155/2012/763248

**Published:** 2012-05-13

**Authors:** Osvaldo Zmener, Cornelis H. Pameijer

**Affiliations:** ^1^Postgraduate Program for Specialized Endodontics, Faculty of Medical Sciences, School of Odontology, University of El Salvador/Argentina, Dental Association, C1113AAC Buenos Aires, Argentina; ^2^University of Connecticut School of Dental Medicine, Farmington, CT 06032, USA

## Abstract

*Objectives*. This retrospective clinical and radiographical study evaluated the 10-year outcome of one-visit endodontic treatment with gutta-percha and a methacrylate resin-based sealer. *Methods*. From an initial sample size of 180 patients, 89 patients with 175 root canals responded to a recall. Treatment outcome was based on predetermined clinical and radiographic criteria. *Results*. Root canals had been adequately filled to the working length in 80 teeth (89.88%), short in 6 instances (6.74%), while 3 (3.37%) with extrusion immediate postoperatively, showed no sealer in periradicular tissues. The difference in the outcomes of treatments with respect to age, gender, preoperative pulp or periapical status, the size of periapical lesions and the type of permanent restorations were not statistically significantly different (*P* > 0.05). Overall, 7 (7.86%) cases were considered clinically and radiographically a failure. A life table analysis showed a cumulative probability of success of 92.13% after 10 years with a 95% confidence interval of 83.0 to 94.0. *Conclusions*. The results of this retrospective clinical and radiographical study suggest that the tested methacrylate-resin based sealer used with gutta-percha performed similarly to other root canal sealers over a period 10 years. *Clinical Implications*. Considering the success rate after 10 years of this methacrylate resin-based sealer can be recommended as an alternative to other commonly used root canal sealers.

## 1. Introduction

It has been reported that after complete debridement and disinfection, total obliteration of the root canal space with biocompatible materials constitutes one of the most important requisites for successful root canal treatment [1]. In this respect, the outcome of the endodontic treatment indicates the extent to which the above conditions has been achieved [2]. Therefore, success or failure rates of treatment modalities are an important part of evidence-based practice in endodontics. Numerous studies [3–6] including a more recent systematic critical review by Ng et al. [7] have been published evaluating the success and failure rates of root canal treatment using clinical and radiographical examination. Although some limitations were reported [2], well-defined predetermined clinical and radiographical criteria are still considered by many authors as a reliable method to evaluate the long-term results of endodontic therapy [3–5, 8–11]; especially as previously has been demonstrated, a good correlation exists between clinical, radiographical, and histological findings [12, 13]. A preliminary short-term retrospective study on 180 patients evaluated the results of root canal treatment of 295 roots filled with laterally compacted gutta-percha cones and EndoREZ sealer (ER, Ultradent Products Inc. South Jordan, UT, USA) [9]. ER is a hydrophilic, 2-component dimethacrylate-based material that meets the essential physicochemical and biological properties required for a root canal sealer according to Pameijer and Zmener [14]. When retreatment is indicated it can easily be removed along with gutta-percha by mechanical instrumentation [15]. After 14–24 months, 145 patients were evaluated for a follow-up examination. An overall success rate of 91% was reported [9]. In a second follow-up study performed 5 years after initial therapy, 120 patients out of 180 were available for follow-up evaluation and an overall success rate of 90% was reported [10]. A third follow-up evaluation also by Zmener and Pameijer [11] of the same patient pool generated 112 patients that were available for examination. After 8 years the overall success rate was 86.5%. As the outcome of the root canal treatment varies over time, the purpose of this retrospective follow-up study was to assess tooth retention based on the success/failure rate of the same patient pool 10 years after root canal treatment.

## 2. Materials and Methods

The protocol for this study was revised and approved by the Ethics Committee for human research of the Argentine Dental Society # 2012-235.

Of the original patient pool attended during 2001–2002, 89 patients (46.07% male and 53.93% female with an age range of 12–75 years) with 175 root canals were available for a 10-year follow-up examination during which they were clinically and radiographically evaluated. Although some of these patients had required further endodontic therapy, only the original treatment of years 2001-2002 was included for evaluation in this study. Subjects were contacted by mail, telephone, or e-mail and invited for a follow-up clinical and radiographic examination Twenty-three (25.84%) patients did not respond to the recall request.

During the initial treatment preoperative radiographs were made, and the status of pulp and periradicular areas was recorded. All treatment had been completed in a single visit by one operator following a precisely defined operative protocol as described by Zmener and Pameijer [9]. Briefly, after an informed consent form was signed by the patient, local anesthesia was administered, a dental dam was placed, and the pulp chamber was accessed. The canals were hand instrumented with a crown-down technique for radicular access combined with a step-back technique for apical preparation. The coronal two-thirds were first flared with #1–3 Gates Glidden drills (Dentsply Maillefer, Ballaigues, Switzerland), and the working length was established with a #15 file, approximately 1 mm short of the radiographic apex. Canal preparation was made with K-type and Hedström files (Dentsply Maillefer) at the apical third to a master apical #30–40 file and coronally to a #60 file. On occasions, the instrumentation sequence was modified due to difficulty in negotiating root canals with complex anatomy. Patency was confirmed with a #10 K-file. Irrigation was performed after every change of instrument using 2.0 mL of 2.5% sodium hypochlorite (NaOCl) followed by rinsing with 2.0 mL of sterile saline. After instrumentation, a final copious rinse with saline was performed. The irrigation solutions were administered with sterile plastic syringes through 30 gauge needles. Excess irrigation solution was removed with sterile paper points; however, the canal walls were kept slightly moist to take maximum advantage of the hydrophilic properties of the resin sealer [16]. The canals were then filled with the EndoREZ sealer delivered through a 30-gauge needle tip (Navitip, Ultradent Products Inc.), followed by lateral compaction of gutta-percha cones. The access cavities were temporized with IRM (Dentsply Caulk, Milford, DE, USA), and the patients were instructed to see their referring dentist for definitive restorative care.

During the follow-up evaluation, a clinical examination was performed and radiographs made. Immediate postoperative and recall radiographs were made using the parallel technique and a Sirona Heliodent unit (Sirona Dental Systems, GmbH, Bensheim, Germany) with a film holder attached to beam-guiding XCP holder (Rinn Corp, Elgin, IL, USA) and Kodak 32 × 43 mm ultraspeed films (Eastman Kodak Company, Rochester, NY, USA). The immediately and 10-year postoperative radiographs were compared in a darkened room using an illuminated X-ray viewer with a magnifying glass. The radiographs were analyzed by two independent calibrated observers, an endodontist with more than 25 years of clinical experience and an experienced radiologist. Calibration was carried out by having the evaluators analyze twice a standard set of 110 individual pairs of postoperative and recall radiographs of endodontic treatments not included in the study that were randomly selected from the files of two private and one postgraduate endodontic service. To meet the inclusion criteria, the radiographs had to be of high quality and had to clearly exhibit periapical tissues, widened periodontal space, loss of cortical bone, changes in trabecular patterns, or easily discernible periapical radiolucencies. When necessary, additional radiographs were made at different horizontal angulations to improve visualization thus improving the reliability of the evaluation. The parameters recorded were number of treated teeth, gender, presence or absence of coronal restoration, periapical radiolucencies, and quality of endodontic treatment. The level of the root canal fillings in relation to the working length was recorded and the quality of the root canal fillings was judged to be adequate when they were placed to the full working length, and no voids were detected while special attention was focused on the last 5 mm of the root canal. Canals that did not meet these conditions were categorized as filled short (>2 mm from the apex), flush or beyond the radiographic apex [17]. Failure of one canal in multirooted teeth was considered a complete failure. In cases with apical radiolucencies, the size of the lesions was estimated on the radiographs as being <2 or >2 mm. Success or failure of the endodontic treatment was determined on the basis of radiographic findings and clinical signs and symptoms according to the criteria listed in Table 1. In cases in which radiographic analysis of periapical status was difficult (questionable cases), teeth were subjected to a limited-volume cone-beam-computed tomography (CBCT) (3D Accuitomo 80, J Morita Corporation, Kioto, Japan). For this purpose the observers were previously calibrated by discussing twice 25 CBCT scans (obtained from a Radiology Institute) that had normal or abnormal periapical findings. If there was a disagreement between the evaluators the X-rays and the CBCT images were reassessed jointly until a consensus was reached.

## 3. Statistical Analysis

Data was statistically analyzed with GraphPad InStat version 3.05 for Windows 95 (GraphPad Software, San Diego CA, USA). The clinical, radiographic, and CBCT data recorded by the two examiners were analyzed for interexaminer agreement. The correlation of treatment outcomes with respect to age, gender, and specific preoperative and postoperative data were analyzed by the Fisher exact test (*P* < 0.05). Taking into consideration, the total number of patients that did not respond to the previous 14–24-month, 5 and 8-year recalls (censored data) [10, 11], a life table survival analysis was used to determine the cumulative probability of success of the 10-year recall. A corresponding 95% confidence interval was determined.

## 4. Results

The examiner calibration showed an interexaminer agreement ratio of 93% revealing a strong interobserver agreement. Therefore, the radiographic and CBCT image interpretation of the results were considered reliable. The recall rate after 10 years (180 patients were originally enrolled) was 49.44%. A total of 89 patients presented for follow-up evaluation. The data collected from the 89 patients were tabulated and the tooth location was noted. The number and location of teeth that were evaluated are shown in Table 2. Distribution of patients by age and gender is presented in Table 3. Distribution by significant preoperative and postoperative factors related to treatment results is shown in Tables 4 and 5, respectively.

**Figure 1:**
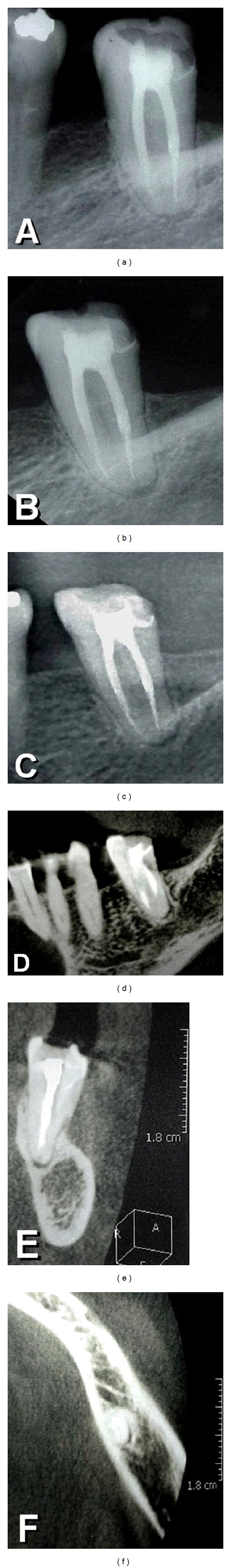
10-year recall radiograph of a left mandibular second molar showing root canals filled with gutta-percha and EndoREZ sealer. (a) Immediate postoperative radiograph of the left mandibular second molar showing root canals filled with gutta-percha and EndoREZ sealer. (b) 8-year recall. (c) 10-year recall. Note the presence of a small residual periapical radiolucent area surrounded by thick cortical bone and normal bone trabeculae. After 10 years the patient was asymptomatic and radiographically the case was evaluated as successful and suggest the presence of an apical scar. (d) Lateral, (e) distal, and (f) occlusal CBCT images confirming the radiographic evaluation.

**Figure 2:**
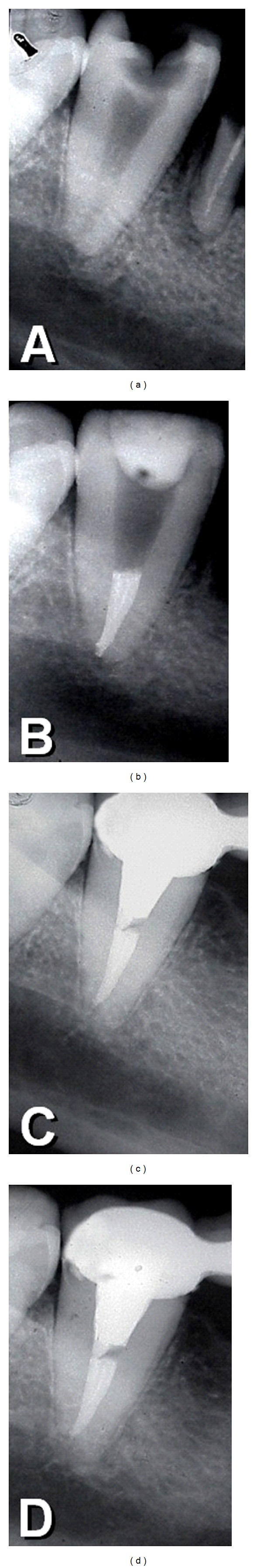
(a) Preoperative radiograph of a mandibular right second molar with deep caries lesion. (b) Immediate postoperative radiograph. Note the root canal filling was partially removed in order to accommodate a post. (c) and (d) 8- and 10-year recall radiographs showing normal periapical tissues.

**Figure 3:**
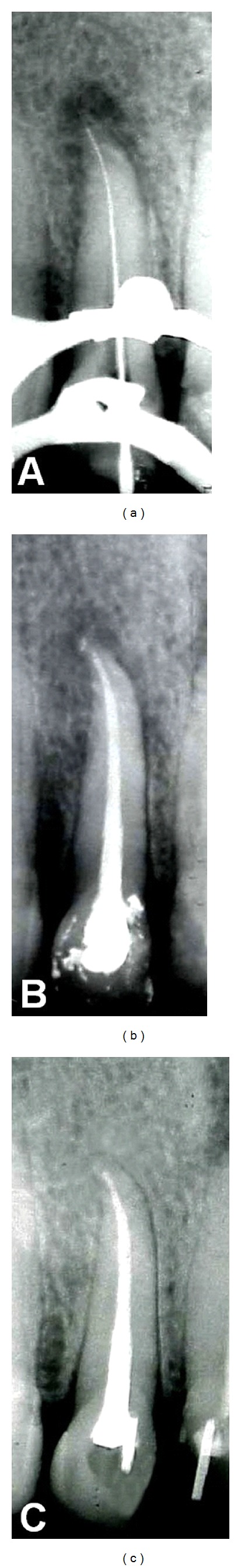
(a) Preoperative radiograph of a maxillary left lateral incisor presenting with a periapical radiolucency. (b) Immediate postoperative radiograph. (c) After 10 years, the recall radiograph revealed that the periapical structures had returned to normal.

After 10 years, 78 teeth (87.64%) were evaluated as adequately filled to the working length. In 5 cases (5.61%) the apical limit of the root filling material was found to be short of the working length. Four (4.49%) of these, which were filled flush at the time of endodontic treatment, underwent resorption of the sealer within the lumen of the canals. These cases showed that the end of the root fill was located at ±1.0 mm from the radiographic apex. Two cases (2.24%), in which extrusion of the sealer was radiographically established immediately after treatment, showed no radiographic evidence of the sealer in the periradicular tissues. Forty-two teeth (47.19%) with preoperative vital pulps were successful in 38 cases while 47 (52.80%) showing preoperative nonvital pulps were successful in 44 cases. Of these cases, five were initially classified as doubtful in which a slight widening of the PDL space was noted. However, these patients were asymptomatic and no fistula or tumefaction was observed. CBCT on these patients confirmed the reliability of the interpretation. A radiograph of a representative case as well as the CBCT images shows normal bone tissues with the presence of well-defined, thick cortical bone. As such, this was considered to be a periapical scar and evaluated as successful (Figure 1). The remaining three cases showed a wide periapical radiolucent area, which was not present at the time of the treatment. Thirty-nine teeth (43.82%) showing preoperative periapical radiolucencies revealed almost total or total healing in 36 cases (Figures 2 and 3), while three presented with some discomfort and showed persistent radiolucencies. These three cases were diagnosed clinically and radiographically as failures. In total, 7 teeth were considered clinically and radiographically a failure. The differences in the outcome of treatments related to age, gender, preoperative pulp or periapical status, the size of periapical lesions, and the type of permanent restorations were not statistically significant (*P* > 0.05). The life table analysis revealed a cumulative probability of success of 92.13% at the 10-year recall with a 95% confidence interval of 83.0–94.0.

## 5. Discussion

This retrospective 10-year clinical and radiographical cohort study performed on the same population as in previous reports [9–11] demonstrated a stable outcome of treatment as defined per parameters outlined by Ørstavik [18]. Using a method evaluating consenting patients and following a predetermined clinical and radiographic protocol is considered a reliable method when evaluating the outcome of endodontic treatment [13, 19, 20]. These evaluation criteria are currently being used by clinicians and are supported by two recent histological investigations [12, 13] that demonstrated a good correlation between radiographic success and the histological status of the periapical tissues in humans.

Twenty-three (25.84%) patients did not respond to the recall. Reasons for declining the recall were lack of interest or time, pregnancy, other general diseases, death, or they had moved elsewhere in the country. Of these 23 patients, three who had the tooth in question extracted because of a root fracture and therefore a recall was a moot issue. All three teeth had a cast post and core and were restored with a crown. The recall rate of 49.44% after 10 years was somewhat below the recall limits established for subject size in clinical trials as reported by Franco et al. [21] but still met the required standards for evidence levels [22]. It was also comparable to previously reported endodontic follow-up studies [5, 6, 15, 21–23] and is in agreement with Ørstavik [18] in that the recall rates in follow-up studies are substantially reduced as the recall period increases. The influence of the recall rates on the results of the current study deserves some discussion. When a patient does not respond to a recall there is always the possibility that one is dealing with a root canal treatment failure and therefore, the data that was generated may not be totally representative of the actual results. It should be noted, however, that the results of endodontic treatments in patients who did not return for followup (censored data) are not considered representative of a particular treatment result category [6]. However, the 23 patients that were not evaluated at this recall were seen at the 8-year follow-up evaluation and categorized as clinically and radiographically successful [11].

Data related to the type and location of teeth was pooled because it has been shown that these factors do not skew the outcome of endodontic treatment [10, 11, 19]. Factors such as gender and age did not negatively affect the results of the study. These observations are in agreement with our previous findings and with those of earlier studies by Selden [24] and Kerekes and Tronstad [25]. Furthermore, no significant differences were found between teeth with vital and nonvital pulps as has been previously reported [4, 19]. The presence of a preoperative apical radiolucency did not appear to adversely affect the outcome of endodontic treatment. This observation is in support of our previous findings [9–11], but contradicts earlier studies by Grossman et al. [23] and others [26, 27], who found significantly lower success rates in teeth presenting with infected root canals and preexisting periapical pathosis. However, our results are in agreement with Sjögren et al. [19] and Peak et al. [28] who showed that the prognosis of teeth with nonvital pulps and preexisting periapical radiolucent areas was as good as that for vital teeth. We can only hypothesize that factors such as early coronal flaring complemented by careful instrumentation with an incremental removal of the bulk of infected root dentine, allowed for a more effective penetration of irrigants, as well as the previously reported tight seal provided by ER [29] may have contributed to a more favorable condition for periapical healing.

At the initiation of the study accidental extrusion of ER was noted in some cases. However, extrusion of the sealer did not show to cause an adverse effect on the outcome of treatments. This observation contradicts the opinion of Seltzer et al. [26], Zmener [30], and Seltzer [31], who stated that extrusion of a root filling material may interfere with the repair process. After 10 years, however, these cases appeared radiographically normal without evidence of sealer in the periapical tissues. These findings suggest that the lack of adverse effects from the extruded ER can be attributed to good tissue compatibility of the sealer, as has been demonstrated in animal studies [30, 32, 33]. In the current study, all patients were treated in a single visit by one operator. Our results tend to support previous evidence that the single-visit endodontic therapy constitutes a reliable procedure [34–37] even in cases with infected root canals and preexisting periradicular pathosis. More recent evidence was provided by Molander et al. [38] and a Cochrane systematic review [39] and demonstrated that the outcome of treatment was not significantly influenced whether root canal therapy was performed in a single visit or multiple visits.

In this study five cases were evaluated as inconclusive. The clinical and radiographic examination suggested the presence of an apical scar surrounded by thick cortical bone. A subsequent CBCT of these cases confirmed the radiographic findings. According to Cotton et al. [40], CBCT visualizes images in the three dimensions rather than in two planes. However, Patel [41] suggested that the CBCT should only be used in situations where the results obtained from conventional radiographs do not allow for a definitive diagnosis. While the CBCT has a lower amount of radiation it is still a source of ionizing radiation to patients [42].

Regardless the methods of observation, clinical observation and interpretation will always be a matter of individual interpretation without reaching complete agreement between individuals [43]. In that respect the interexaminer agreement of 93% in this study is quite acceptable.

In the current study, 49.43% of the recalled patients presented with a post in one or two root canals had a success rate of 44.94%, while 50.56% presented with single metal/ceramic, amalgam, and resin composite or glass ionomer coronal fillings with a success rate of 47.19%. At recall these patients were asymptomatic without radiographic changes in the periapical tissues. These results are in agreement with previous studies [6, 19] in which it was reported that the type of coronal restoration (single coronal restoration, presence, or absence of a post in the canal) did not significantly affect the outcome of endodontic treatment.

## 6. Conclusion

Within the limitations of this clinical and radiographic study the results suggest that ER used in conjunction with gutta-percha constitutes an acceptable root canal filling procedure. Patients recalled after 10 years reported being comfortable and the treated teeth continued to be functional. The sealer appeared to be well tolerated by periapical tissues even in cases of accidental extrusion beyond the apical foramen. Furthermore, the success rate was comparable to what has been reported in the literature for different sealers.

## Figures and Tables

**Table 1:** Criteria for clinical and radiographic interpretation of success and failure.

Outcome of treatment	Clinical and radiographical findings at recall
Success	(1) Radiographically, the contours and width of the PDL space were within normal limits or slightly widened around an accidental overfill and the patient was free of symptoms. Slight tenderness to percussion for a brief postoperative period was considered acceptable. (2) The size of a preoperative radiolucent area decreased by at least 50%, and the patient was free of symptoms, or the contours and width of the PDL space had returned to the normal. (3) Absence of preoperative periapical radiolucency which remained unchanged over time.

Failure	(1) Periapical radiolucency was observed in the preoperative radiograph and remained unchanged or increased in size over time. (2) A root in absence of preoperative periapical pathosis developed a radiolucency over time.

**Table 2:** Tooth number and location of teeth in the maxillary and mandibular arch evaluated 10 years postoperatively.

	Maxillary	Mandibular	Total
Central incisor	16	1	17
Lateral incisor	8	1	9
Canine	8	3	11
First premolar	4	6	10
Second premolar	6	7	13
First molar	6	11	17
Second molar	4	5	9
Third molar	1	2	3

Total	53	36	89

**Table 3:** Outcome of treatment by gender and age in root canals filled with gutta-percha and ER after 10 years.

Factor	# of cases %	Success %	Failure %
Gender			
Male	41 (46.07)	37 (41.57)	4 (4.49)
Female	48 (53.93)	45 (50.56)	3 (3.37)
Age			
12–30	11 (12.35)	9 (10.11)	2 ( 2.24)
31–55	57 (64.04)	54 (60.67)	3 (3.37)
56–75	21 (23.59)	19 (21.34)	2 (2.24)

**Table 4:** Relationship of preoperative factors to treatment results in root canals filled with gutta-percha and ER.

Factor	# of cases %	Success %	Failure %
Pulp diagnosis			
Vital	42 (47.19)	38 (42.69)	4 (4.49)
Non vital	47 (52.80)	44 (49.43)	3 (3.37)
Periapical radiolucency			
Present	39 (43.82)	36 (40.44)	3 (3.37)
Absent	50 (56.17)	46 (51.68)	4 (4.49)
Lesion size			
<2 mm	35 (39.32)	32 (35.95)	3 (3.37)
>2 mm	4 (4.49)	2 (2.24)	2 (2.24)

**Table 5:** Relationship of final restoration to treatment results in root canals filled with gutta-percha and ER.

Restoration	# of teeth %	Success %	Failure %
None	—	—	—
Post (with or without crown	44 (49.43)	40 (44.94)	4 (4.49)
Coronal filling (amalgam, composite, glass ionomer, etc.)	45 (50.56)	42 (47.19)	3 (3.37)
